# Feasibility Study for a Randomized Controlled Trial of Aromatherapy Footbath for Stimulating Onset of Labor in Term Pregnant Women

**DOI:** 10.3390/ijerph22060950

**Published:** 2025-06-17

**Authors:** Yuriko Tadokoro, Kaori Takahata

**Affiliations:** 1 Chiba Faculty of Nursing, Tokyo Healthcare University, Funabashi 273-8710, Japan; 2 Chiba Postgraduate School of Nursing, Tokyo Healthcare University, Funabashi 273-8710, Japan; 3 School of Nursing, Shonan Kamakura University of Medical Sciences, Kamakura 247-0066, Japan; k.takahata@sku.ac.jp

**Keywords:** pregnant women, aromatherapy, footbath, complementary alternative medicine, stimulation of labor, feasibility study

## Abstract

We evaluated the feasibility of a new research methodology designed for conducting a future, large-scale randomized controlled trial (RCT). This future RCT is aimed at evaluating the effects of repeated aromatherapy footbaths on stimulating the onset of labor. Herein, we conducted a pilot RCT with two arms among low-risk pregnant women at or beyond 39 weeks of gestation before labor onset. These two arms consisted of a treatment group performing aromatherapy footbaths twice a day (n = 7) and a usual care group (n = 8). This study was prospectively registered in the Clinical Trials Registry of the University Hospital Medical Information Network in Japan (UMIN000037398). Feasibility was assessed across the domains of *acceptability*, *demand*, *implementation*, *practicality*, *process*, *resources*, and *management* using questionnaires, researcher records, and semi-structured interviews with the treatment group and midwives at the setting facility. The new research methodology was found to be feasible, although challenges were identified in the *process* and *implementation*. For *process*, the research participation rate was 55.5%. For *implementation*, the adherence rate among the multiparous participants in the treatment group ranged from 50% to 94%. An imbalance between both groups was found. Areas that need careful planning and methodological improvements include random allocation, treatment method, and participation criteria.

## 1. Introduction

Full-term delivery between 39 and 40 weeks of gestation has the best neonatal outcomes particularly for respiratory morbidity [1]. To avoid delivery at or more than 41 weeks of gestation, labor is induced in approximately 23% of pregnancies [2]. Although medical labor induction aims to mitigate neonatal risks, it has its own complications, including a higher incidence of cesarean sections [3], uterine rupture [4], and heightened labor pain [5]. Notably, a substantial proportion (59.2%) of women who have undergone medical labor induction express a desire to be informed of complementary and alternative medicine (CAM) approaches for labor stimulation [6].

Pregnant women who wish to have a healthy and normal delivery adopt various CAM modalities. In the USA, CAM is adopted by 28% of pregnant women [7]. Among the CAM modalities, aromatherapy has gained attraction from pregnant women and medical staff for stimulating and augmenting labor [8,9,10,11,12,13,14]. Aromatherapy involves the use of essential oils extracted from various plants. These are administered through inhalation of the essential oil’s scent; baths diluted with the essential oils for full body, foot, or hand immersions; or massage with massage oils diluted with essential oils [15]. Although anecdotal evidence and practices support the use of aromatherapy in this context, rigorous scientific evaluation of the effects of aromatherapy remains limited [14,16].

Vaginal delivery is achieved by labor which is induced by uterine contractions. Uterine contractions are initiated by the hormone oxytocin. In a previous study, salivary oxytocin level was shown to be significantly increased after inhalation of clary sage or lavender essential oils in postmenopausal women [17]. Moreover, the repeated use of lavender essential oil for labor augmentation was found to shorten the duration of labor and delivery [18,19,20], which requires uterine contractions that is induced by oxytocin. In our previous study [14], we found that an aromatherapy footbath containing the essential oils of clary sage and lavender significantly increased salivary oxytocin level in term pregnant women, whereas a plain footbath not containing any essential oils showed a decreasing trend in the salivary oxytocin levels. Nevertheless, the changes in the salivary oxytocin levels before and after the footbath were not significantly different between the aromatherapy footbath and the plain footbath. The lack of a significant difference is suggested to be caused by the large standard deviations in the salivary oxytocin levels owing to the lack of an extraction step in the measurement method. Although the standard deviations in the salivary oxytocin level in our previous study did not exceed the standard deviations in other previous studies [21,22], the layering of deviations in the salivary oxytocin level resulted in a lack of a significant difference. The exact amount of increase in the salivary oxytocin level needed to induce the onset of labor remains unclear. In another study, breast stimulation as another CAM to induce labor resulted in an increase in the salivary oxytocin level similar to the increase induced by aromatherapy footbath, and that repeated stimulation increased the oxytocin level [23].

Altogether, the repeated use of aromatherapy can increase the oxytocin level and uterine contraction, subsequently stimulating labor. Therefore, based on our previous study of aromatherapy footbath [14], we recommended the evaluation of the repeated use of aromatherapy footbath for stimulating labor in a larger sample size with targeted clinical outcomes in a randomized allocation (i.e., larger RCT). Herein, the present feasibility study holds potential in validating the effects of the repeated use of aromatherapy footbath on labor stimulation. The accumulation of robust evidence-based data can be used to empower pregnant women facing beyond full-term and post-term pregnancies with informed choices regarding evidence-based labor management strategies. We found no adverse events in our previous study. Thus, we could confirm in our previous study that our one-time aromatherapy footbath was preferred by pregnant women as the withdrawal rate was very low at 1.2%, implying acceptance of the method by pregnant women. Our previous aromatherapy footbath method was administered in a study setting and prepared by researchers. As for the evaluation of the repeated use of aromatherapy footbaths, the aromatherapy footbath needs to be prepared by the participants themselves and administered in the participants’ home for their convenience. However, the feasibility of such method remains unknown.

Prior to conducting a large randomized controlled trial (RCT), it is recommended to carry out a feasibility study of the new methods [24,25,26]. Therefore, we conducted the present study to rigorously evaluate the feasibility of a new research methodology designed for conducting a future, large-scale RCT aimed at assessing the effects of the repeated use of aromatherapy footbaths on stimulating the onset of labor. Specifically, we will assess whether the following key domains are satisfied to determine the feasibility of subsequent definitive trials: *acceptability*, *demand*, *implementation*, *practicality* [24], *process*, *resources*, and *management* [26]. The present study refines these feasibility domains systematically and facilitates their identification for optimizing a future RCT design and the procedures involved. This allows saving unnecessary human resources and financial cost [26].

## 2. Materials and Methods

### 2.1. Study Design and Setting

We used a pilot RCT design with two parallel arms: *a treatment group* performing 20 min aromatherapy footbaths and a usual care group for comparison. The participants were recruited at a single birth center located in a metropolitan area in Japan between July 2019 and March 2020. This study was prospectively registered in the Clinical Trials Registry of the University Hospital Medical Information Network in Japan “https://center6.umin.ac.jp/cgi-open-bin/ctr/ctr_view.cgi?recptno=R000042638 (accessed on 12 June 2025)”, with initial registration completed on 17 July 2019. This work was designed to carry out a feasibility study of a new research methodology for conducting a future, large-scale RCT to assess the effects of the repeated use aromatherapy footbaths on stimulating the onset of labor.

### 2.2. Pilot Randomized Controlled Trial

#### 2.2.1. Participants and Sample Size

The inclusion criteria were low-risk pregnant women aged 20 years or older who were planning to have a vaginal delivery, who were at least at 39 weeks of gestation but had not yet experienced the onset of labor, and without any obstetric risks such as multiple gestations, a history of cesarean section or stillbirth, and pregnancy complications including pregnancy-induced hypertension, gestational diabetes, or fetal diseases. The exclusion criteria were pregnant women with (1) olfactory disorders, (2) mental illness, or (3) known allergies to plants, aromatherapy products, or multiple foods or drugs. Women who had olfactory disorders (1) can have impairment in the recognition of essential oil scents and were excluded. Women who had mental illness (2) were excluded to avoid unnecessary psychological burden caused by research participation. Additionally, to minimize the risk of allergic reactions potentially induced by the research procedure, women who had known allergies (3) were excluded. A researcher or a research assistant confirmed (1), (2), and (3) during research explanation. Given that the intervention commenced at *39 weeks and 0 days of gestation* in the treatment group, recruitment of pregnant women was conducted before reaching this gestational age.

A patch test of the essential oils used in the treatment group was applied to the participants after agreeing to participate in this study. The participants were checked for any allergic reactions 15–20 min and 72 h after the application of the essential oils. If there was a positive reaction, research participation was discontinued.

The present study did not require a sample size calculation, being a feasibility study [26]. Nevertheless, as other feasibility studies have indicated, a sample size of 6 to 15 participants per group was deemed sufficient to obtain adequate data for a feasibility analysis [27,28,29,30,31,32,33,34]. Recruitment was planned to be closed upon reaching this target sample size or when sufficient interview data on the feasibility of the new research method were collected.

#### 2.2.2. Randomization and Masking

Eligible participants were randomly assigned in a 1:1 ratio to either the treatment group or the usual care group with simple randomization using a web-based randomization system “https://autoassign.mujinwari.biz (accessed on 12 June 2025)”. Following randomization, a detailed explanation of the assigned intervention was provided by a researcher or a research assistant. Blinding of the participants was not possible because of the nature of the intervention. Masking of the staff at the setting facility was not implemented in this study, but will be carried out in future studies. The masking will be conducted by limiting the information shared with the facility staff to whether an individual is participating in the study, and asking the facility staff and the participants not to confirm the participants’ assignment from each other.

#### 2.2.3. Intervention

**Figure 1:**
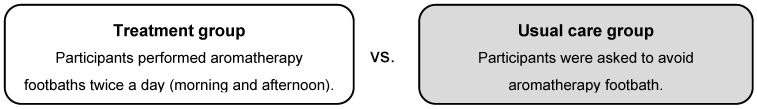
Interventions in the treatment group and the usual care group.

**Figure 2:**
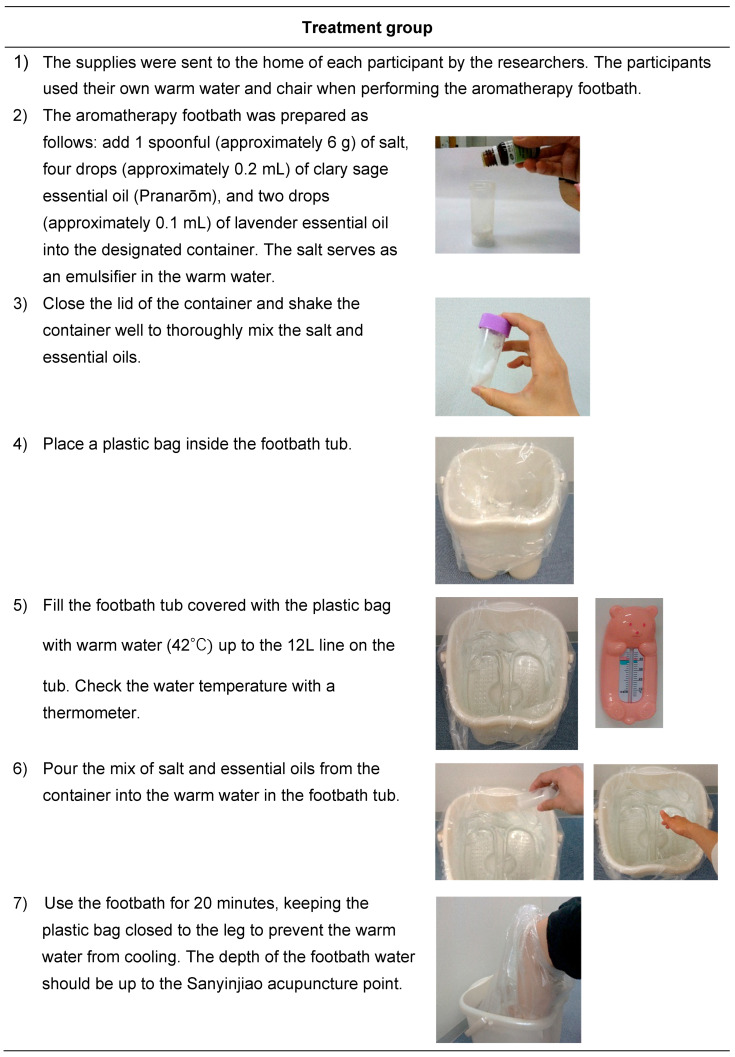
Procedure of the treatment group.

The participants in both the treatment group and the usual care group received the same standard care. Figure 1 shows the intervention difference between the treatment group and the usual care group which commenced from 39 weeks and 0 days of gestation to the onset of labor. Figure 2 shows the procedure of the treatment group from preparation to the use of aromatherapy footbaths. The footbath procedure followed the steps in our previous study [14]. However, each footbath was prepared, performed, and cleaned by the participants themselves at their home and not by researchers at a study setting. After completion of the footbath, the participants dumped the warm water out of the footbath tub over the plastic bag. The participants did not need to wash the footbath tub with a bath detergent as long as the footbath water splashed on the footbath tub. A new plastic bag was used with each footbath.

#### 2.2.4. Data Collection

Following the planned future RCT, we similarly collected outcome data, adverse events, basic characteristics, and intervention adherence. As for the outcome data, *labor induction*, *delivery week*, *labor and delivery duration*, *labor augmentation*, and *delivery mode* were extracted from the medical records. As for the adverse events, allergic reactions of the skin when soaked in the footbath were monitored in the treatment group [35,36]. The participants in the treatment group were instructed to contact the midwives of the setting facility and then the researchers, and to discontinue further aromatherapy footbaths if they experienced any skin symptoms. Data on the basic characteristics and intervention adherence were collected using a self-administered questionnaire or medical records. This questionnaire was provided to the participants in both the treatment and usual care groups after the group assignments. They were asked to complete and return the questionnaire by postal mail within 40 days postpartum.

#### 2.2.5. Analysis

The present study focused on evaluating the feasibility of a new research methodology for conducting a future, large-scale RCT. Therefore, statistical analysis was not necessary [26,37]. For the future RCT, the outcomes between the treatment and usual care groups will be compared using statistical software R for Windows.

### 2.3. Investigation of Feasibility

#### 2.3.1. Data Collection for Investigating Feasibility

For the present assessment of *feasibility*, it was recommended to use a multifaceted approach [38], incorporating quantitative and qualitative research methods [24,31,39]. We used both quantitative and qualitative research methods to collect data regarding feasibility. The feasibility of the new research methodology was investigated across the following domains: *acceptability*, *demand*, *implementation*, *practicality* [24], *process*, *resources*, and *management* [26]. A detailed overview of the data collection, data collection timing, and data collection methods for each feasibility domain is described by Andreassen et al. [31] in Table 1.

Data collection methods included the use of self-reported questionnaires from both the treatment and usual care groups, research records by the researchers, and semi-structured interviews. The interviews were administered individually in a semi-structured format and lasted for approximately 30 min with the participants in the treatment group within two months postpartum and with the midwives in the setting facility within one month after the completion of data collection for the pilot RCT. All interviews were recorded and transcribed.

#### 2.3.2. Feasibility Evaluation

Quantitative data obtained from the research records by the researchers and self-reported questionnaires were shown in descriptive statistics. Qualitative data obtained from the interviews and self-reported questionnaires were extracted and categorized by content representing each feasibility domain. Based on the findings from both the quantitative and qualitative data, the feasibility of a future RCT was assessed in terms of participants and the setting perspectives. Identified areas for improvement were also documented [26]. A future RCT would be assumed feasible if the following criteria were met: *acceptability*, at least half of the participants in the treatment group respond that they will perform the footbath treatment in their future pregnancy and they would recommend the footbath treatment to other pregnant women; *process*, the target research participation rate of 70% or above is met; and *resources*, the follow-up rate of 80% or above is met [26,40].

### 2.4. Ethics

This study was conducted in accordance with the ethical standards set forth in the Helsinki Declaration of 1975 with approval by the Ethics Committee on Human Research of Tokyo Healthcare University (Approval No.: 31-4A). Written informed consent was obtained from all the participants. The participants in the treatment group received a compensation for their time and effort amounting to ¥5000 for completing the interview and returning the questionnaire, or ¥2000 for only completing and returning the questionnaire. The participants in the usual care group received a token of appreciation.

## 3. Results

**Figure 3:**
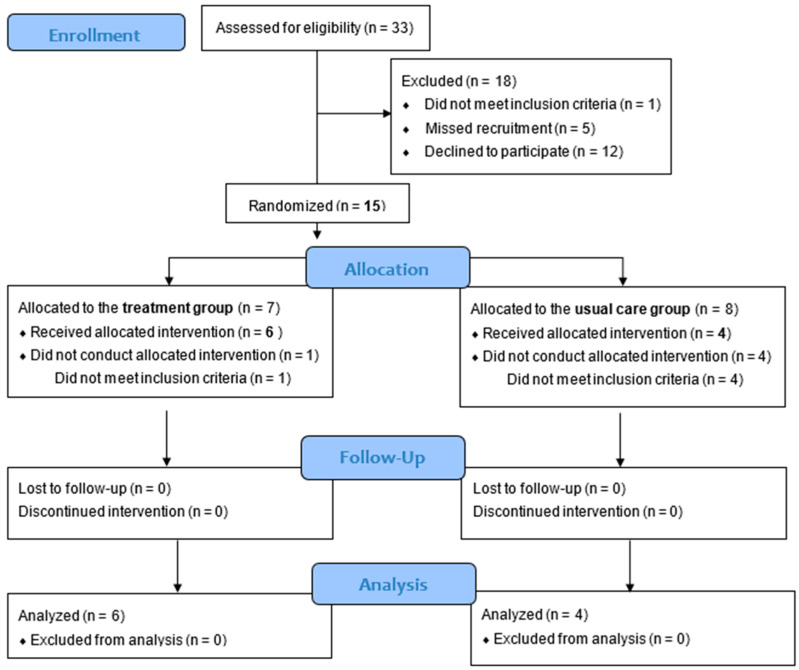
Flowchart showing intervention profile. n: number of participants.

Figure 3 shows the flow diagram of the participants from enrollment to analysis in the present pilot RCT following the CONSORT 2010 statement: extension to randomized pilot and feasibility trials [25]. Table 2 shows the basic characteristics of each group. Table 3 shows the outcome data collected following a pilot RCT method. There were no participants with positive allergic reactions in the patch test or participants with skin symptoms in the treatment group. Changes in the methods were not performed after commencement of the study. The interviews were successfully conducted with all six women in the treatment group who completed the intervention and with two midwives at the setting facility. The recruitment was closed when the interviews with seven women were anticipated to be obtained in the treatment group. The subsequent sections detail the findings for each feasibility domain.

### 3.1. Acceptability

#### 3.1.1. Acceptability Among Pregnant Women

Satisfaction with the treatment was assessed using a scale ranging from one (*not satisfied*) to five (*most satisfied*). Five out of six women in the treatment group rated their satisfaction as either four or five, with the remaining women rating it as three. All the six women in the treatment group indicated that they would be willing to perform the treatment in their future pregnancies, citing feelings of relaxation and comfort. However, regarding recommending the treatment to other pregnant women, two of the six women expressed willingness, whereas four of the six women expressed a neutral stance, citing potential individual preferences for essential oil scents. They indicated that they would only share their experiences of the treatment as a suggestion for pregnant women, not as a recommendation.

#### 3.1.2. Acceptance Among Staff in the Setting Facility

The midwives in the setting facility perceived that aromatherapy footbaths aligned well with the facility’s culture. They highlighted the following advantages of conducting the aromatherapy footbaths within the facility: the intervention provided an opportunity to consider the implementation of aromatherapy into the facility’s care and it allowed multiparous women in the treatment group to have a break from childcare and household responsibilities by having their own time. However, the midwives had a feeling of regret for the participants who were interested in performing the aromatherapy footbath but were assigned to the usual care group.

### 3.2. Demand

Before randomization, all 15 participants answered the questions regarding *demand*. Eleven participants (73.3%) expressed interest in the aromatherapy footbath, eight participants (53.3%) in stimulating the onset of labor, and four participants (26.6%) in stimulating the onset of labor through the aromatherapy footbath. Three participants (20.0%) indicated their intention to perform the treatment regardless of their study involvement.

### 3.3. Implementation

The intervention periods in the treatment group were varied from two days to nine days. Only one primiparous woman who had a two-day of intervention period was able to follow the treatment. Between 50% and 94% of the other women who were all multiparous were able to perform the planned aromatherapy footbath. The primary challenge for following the treatment was the difficulty in adjusting their schedules while taking care of their older children. However, their partners provided assistance by caring for the older children. The participants in the treatment group reported that *promotion* (relaxation and sleep) and *desire* (early delivery) motivated them to adhere to the treatment.

### 3.4. Practicality

All women in the treatment group found the method for preparing and cleaning the aromatherapy footbath to be feasible. Several suggestions for improvement were identified. These included using a container with a wider opening for easier salt addition, utilizing a mobile footbath tub with a drainage function to address the weight of the tub filled with water, and providing clearer instructions on maintaining water temperature and suggestions for activities during the 20 min treatment session.

Half of the women in the treatment group reported that they could perform the treatment independently by purchasing the necessary items from physical or online stores. The other half indicated that they could not perform the treatment independently because of a lack of knowledge about where to purchase the items. There were no unexpected additional costs incurred in the treatment group. The estimated cost for research execution in a future RCT was ¥13,000 for each participant in the treatment group, which covers purchasing and transportation of the treatment items, postage for returning the questionnaire, and monetary compensation. The estimated cost was ¥200 for each participant in the usual care group, which covers postage for returning the questionnaire and a token of appreciation.

### 3.5. Process

Of the thirty-three pregnant women initially assessed for eligibility, one woman was excluded for not meeting the inclusion criteria and five women were missed as they could not be provided an explanation of the study during the recruitment. Among the remaining 27 women recruited, 12 women declined to participate and 15 women participated and were randomized in the study, resulting in a participation rate of 55.5%. After the randomized allocation to each group, five participants (33.3%) in both groups could not continue the study because they no longer met the inclusion criteria due to the onset of labor before 39 weeks of gestation.

### 3.6. Resources

In the case of *resources*, although the follow-up rate based on outcomes for a future RCT was 100%, the return rate for the questionnaire was 90%, that is, nine out of ten participants. The eligibility criteria for the participants were clear, with no ambiguous wording in judging the criteria and no unnecessary criteria. All the participants in the treatment group reported that the time required to complete the questionnaire was within 1–5 min upon their acceptance. They also indicated a clear understanding of all the study documents including the research explanation, instruction for the treatment, and questionnaire. There were no missing values or unintended responses in the questionnaire and data collection.

### 3.7. Management

The explanation during the recruitment took a maximum of 30 min for each participant. There were no questions from the participants outside the recruitment explanation during the study period. All supplies for the treatment group were prepared at the start of the study and sent to each participant, the handling of which took a maximum of 5 min for each participant. There were no issues in the coordination of the research supplies.

The midwives at the setting facility were asked to select the candidate participants for the study, contact the researchers, and export the medical records, which were all successfully completed. The midwives reported no issues in collaborating with the present study. They indicated their ability and willingness to collaborate in a future RCT. The outcomes of the pilot RCT could be assessed using R-4.4.2 for Windows.

## 4. Discussion

We conducted this study to investigate the feasibility of a new research methodology designed for conducting a future, large-scale RCT. The feasibility domains that we investigated were *acceptability*, *demand*, *implementation*, *practicality* [24], *process*, *resources*, and *management* [26]. The study methods were shown to be feasible for the participants and staff in the setting facility. However, some of the domains for evaluating the feasibility by the researchers, namely, *acceptability* and *process*, needed further improvements.

Regarding *acceptability*, the pre-set criteria were met, wherein at least half of the participants in the treatment group would express their desire to use the treatment again in their next pregnancy. Relaxing effect and comfort, the subjective benefits provided by the treatment, are thought to have brought about the satisfaction and the desire. Other CAM methods aimed at stimulating labor, such as breast stimulation [41], acupuncture, and acupressure [42] are also possible options for pregnant women. Aromatherapy footbaths are easier and more comfortable to perform than other options, and if the method of implementation and evidence of benefits are established, aromatherapy footbaths will be especially beneficial for pregnant women.

*Acceptability* at the facility is also feasible because the treatment fits within the facility’s culture and provides an opportunity to expand the options for care provided at the facility. On the other hand, the midwives were apologetic to some participating women who were assigned to the usual care group and were unable to perform the treatment. In future studies, this psychological burden to midwives can be reduced by masking the allocation to the staff.

Regarding *demand*, over half of the participants expressed interest in labor stimulation and aromatherapy. Other surveys have reported that 15.2% of women used aromatherapy during pregnancy [43]. However, the percentage of expressing interest only and that of actually using the treatment are different. A steady point is that pregnant women indicated a consistently high demand for aromatherapy.

Regarding *practicality*, following the treatment method was shown to be feasible for pregnant women. Furthermore, we could obtain suggestions for improvements on the footbath items, which could only be realized when conducting a feasibility study. In terms of obtaining the footbath items for the participants, it was found that the researchers need to prepare the treatment items or provide complete information on where to purchase those items in future studies. The cost will inevitably be higher in the treatment group than in the usual care group because essential oils are expensive. The present information on costs will contribute to the development of more accurate plans for future studies.

Regarding *resources*, the pre-set follow-up rate of 80% or above was achieved in this study. This follow-up rate is equivalent to the follow-up rate in previous studies [32,40]. The results showed that the explanations of the inclusion criteria, outcome data collection, and documents and questionnaire for the participants in the study methods were considered to be appropriate.

Regarding *management*, since there were no additional questions from the participants, the researchers were not required to respond unexpectedly. In addition, the treatment items were sent smoothly. This study was carried out very smoothly that the midwives in the setting facility also expressed their intention to cooperate in any future study. Moreover, there were no participants who had allergic reactions during the recruitment or treatment.

We recognized some issues regarding the following three items: *implementation*, *acceptability*, and *process*. First, in terms of *implementation*, the adherence rate was low among the participants in the treatment group, particularly among multiparous women. However, the adherence rate was not that low compared with the adherence rates from other feasibility studies [27,29,44,45]. In a previous study involving 15 participants who had diabetes, a low adherence rate of 24% was shown when interventions of two to three muscle-strengthening exercises per week were implemented for eight weeks, and an adherence rate of 48% was found when interventions of at least 150 min of aerobic exercise per week were implemented for 12 weeks [27], which imply that a smaller frequency of interventions results in a higher adherence rate. We set the frequency of aromatherapy footbaths twice a day, as the maximum frequency of actual usage [13]. In previous reports, the frequency was varied from multiple use a day to once a week [10,46]. In reference to a previous study showing that repeated breast stimulation increased salivary oxytocin level as CAM to stimulate labor, the procedure was conducted once a day [23]. Therefore, the reduction in the frequency of conducting aromatherapy footbaths to once a day can be considered as a feasible countermeasure. Alternatively, as primiparous women are more likely to exceed their due date than multiparous women [47,48], there can be a plan to conduct research focused on primiparous women who more likely need stimulating the onset of labor without changing the intervention.

Second, in terms of *acceptability*, the pre-set criterion that at least half of the pregnant women would be willing to recommend the treatment to other pregnant women was not met. It is important to note that the pregnant women who were not willing to recommend the treatment only took a neutral stance to respect individual preferences, which did not deny the *acceptability* of the treatment among pregnant women. Therefore, it is necessary to reconsider the feasibility criteria in on the area of individual preferences.

Third, in terms of *process*, the participation rate of 55.5% did not meet the pre-set target of 70% or above. In previous feasibility studies, the participation rates were reportedly between 52.4% and 66.4% [32,40,49]. In another study where aromatherapy footbaths were conducted twice on separate days for term pregnant women, the participation rate was 56.9% [14]. Compared with the participation rates from previous studies, our participation rate was not low. On the other hand, because of the nature of the intervention, it is impossible to assign participants before 39 weeks of gestation to each group. Consequently, after the randomized allocation, 33% of the participants went into labor before the start of the intervention and were thus excluded based on the eligibility criteria. Another potential consideration is that multiparous women could expect to have difficulties in making schedule adjustments, as shown in the results of *implementation*, which can contribute to the low participation rate. These items cause an imbalance in the number of participants between the treatment group and the usual care group. Therefore, considering that the participants will be excluded after the assignment, a higher participation rate is essential in a future study. To increase the number of participants, to achieve full-term delivery, and to achieve balanced evaluation, further modification of the intervention and a random allocation can be considered. The other CAM options of breast stimulation, acupuncture, and acupressure for stimulating labor were previously used in pregnant women over 36 weeks of gestation [41,42] or 38 weeks of gestation [23]. Considering all together that CAM treatments have accumulated effects [14] and that full-term delivery between 39 and 40 weeks of gestation has the best neonatal outcomes [1], the treatment has to be started before term-pregnancy. Therefore, 37 or 38 weeks of gestation can be a better timing for starting the intervention. Considering the imbalance in the analyzed number of participants between the two groups and the low adherence rate in multiparous women, a randomized allocation for the stratification of primiparous/multiparous women and gestation weeks of pregnancy should be conducted. Furthermore, a future RCT can involve multiple facilities to collect outcomes from a larger number of participants, which will also require the stratification of the participants among the facilities.

### Strengths, Limitations, and Future Research Directions

The strength of this study is that it followed the methodological tutorials and guides for conducting feasibility studies based on existing feasibility studies, as reported previously [24,26]. Therefore, it can serve as a methodological reference for enhancing future feasibility and CAM studies. The limitations of this study are fourfold. *First*, unlike a future RCT, the staff at the setting facility were not masked. In the feasibility domain of *management*, the intention to participate in a future study by the staff at the setting facility may have differed depending on whether masking was performed. *Second*, this study was conducted in a single facility. Accordingly, the judgment that *acceptability* and *management* were feasible by setting might differ depending on facility culture and circumstances. *Third*, the usual care group did not carry out aromatherapy footbaths without the essential oils to save costs and maintain research execution. A better future design for conducting the aromatherapy footbath in the usual care group is to follow the same instructions but without the essential oils. *Fourth*, all the feasibility domains were not investigated. The feasibility domains that were not investigated include *adaptation*, *integration*, *expansion*, *limited efficacy* [24], and *scientific effects* [26]. In particular, an examination of *limited efficacy* requires more participants than the participants of this study. Before investigating the rest of the feasibility domains, planning to improve the study method has been conducted using the following approaches: conducting stratified randomization, focusing on primiparous women as participants, early commencement of the intervention, less frequency of aromatherapy footbaths in the treatment group, easier handling of the footbath tub, and clearer instructions during the treatment. Furthermore, we should strictly determine the number of participants to be recruited and the recruitment period based on the percentage of the participants who will be excluded after the assignment.

A feasibility study is conducted to evaluate the methodology of an RCT before performing the RCT, which might not show direct and new clinical medical knowledge. The feasibility study, particularly in the first step, can use the qualitative data of subjective perception that is not directly linked to medical outcomes [24,26,28,31,45,50]. Moreover, the feasibility study method does not estimate treatment effects because such estimate may be unrealistic and biased due to the limited number of participants [26]. A feasibility study with a small number of participants should not lead to conclusions regarding the effects of the treatment and such a study can have possible variations among the participants. However, the number of participants of the present feasibility study is not smaller than that of other feasibility studies [27,28,29,30,31,32,33,34]. The findings identified in the present feasibility study can serve as a foundation for refining the next steps of a future feasibility study. In the subsequent research phases, careful attention has to be given to this possible variation while investigating the remaining feasibility domains. An initial feasibility study facilitates the identification of better study methods and leads to a more feasible, larger, and rigorous RCT in the future.

Going another step further is the comparison of different modalities to induce labor. Breast stimulation as one modality for inducing labor, categorized under CAM, has shown cumulative effects in increasing oxytocin levels [23]. Given its efficacy, an RCT could be conducted to compare the outcomes of repeated use of breast stimulation alone versus a combination of aromatherapy footbaths and breast stimulation.

## 5. Conclusions

This study confirmed the feasibility of a new research methodology designed for conducting a future, large-scale RCT for assessing the effects of the repeated use aromatherapy footbaths on stimulating the onset of labor in terms of *acceptability*, *demand*, *practicality*, and *resources* as feasibility domains. For the *process* and *implementation* domains, our findings suggest the need for further improvements particularly in terms of making random allocation, the commencement timing and treatment frequency, and the participation criteria to achieve higher adherence and participation rate. A second feasibility study with modifications based on this present feasibility study to investigate the rest of feasibility domains including limited efficacy is warranted.

## Figures and Tables

**Table 1:** Data collected for investigating the feasibility of the new research methodology and collection methods.

Feasibility Domain	Data Collected	Data Collection Timing	Data Collection Method
***Acceptability*** *To what extent is the treatment judged as suitable, satisfying, or attractive to pregnant women and setting staff?*	Satisfaction with the treatment Preference for conducting the treatment for future pregnancy Whether participants would recommend the treatment to pregnant women	After delivery	Interview with the participants in the treatment group
	Sense of adaptation with the facility’s culture Advantages/disadvantages of conducting the intervention in the facility		Interview with the midwives of the facility
***Demand*** *To what extent is the treatment likely used?*	Interest in and intention for conducting the treatment	Before randomization	Records from oral questions to the participants by researchers
***Implementation*** *To what extent can the treatment be successfully delivered to pregnant women?*	Degree of execution	After delivery	Self-reported questionnaire from the participants in both groups
	Success or failure of execution Resources needed to implement Factors affecting implementation ease or difficulty		Interview with the participants in the treatment group
***Practicality*** *To what extent can the treatment be carried out with pregnant women using existing means, resources, and circumstances and without outside intervention?*	Ability of the treatment group to carry out the treatment Whether the treatment could be performed without supplies provided	After delivery	Interview with the participants in the treatment group
	Financial costs of research execution		Records by researchers
***Process*** *Key to success*	Research participation rate (within the recruited eligible women who consented to participate/recruited eligible women)	Before randomization	Records by researchers
***Resources*** *Time and resource problems*	Follow-up rate	After delivery	Data from medical records by researchers and self-reported questionnaire from the participants in both groups
	Appropriateness of the eligibility criteria for participants		Records by researchers
	Understanding of documents including research explanation, instructions for the treatment, and questionnaire Time required to complete the questionnaire		Interview with the participants in the treatment group
	Missing values or unintended responses in questionnaire		Self-reported questionnaire from the participants in both groups
***Management*** *Potential human and data management problems*	Overload of the study execution for researchers Handling of research supplies Required assistance availability by facility Capturing data for pilot RCT using software	After delivery	Records by researchers
	Facility willingness and capacity to collaborate Issues being raised in a facility		Interview with midwives of the facility

RCT: randomized controlled trial. Self-reported questionnaires from both the treatment and usual care groups and data from research records by the researchers were collected in the present pilot RCT.

**Table 2:** Basic characteristics of the participants for each group.

	Treatment Group (n = 6)	Usual Care Group (n = 4)
Age (years)	34.3 (*5.4*)	35.7 (*3.9*)
BMI before pregnancy (*SD*)	22.2 (*4.7*)	20.4 (*1.4*)
BMI at delivery (*SD*)	26.0 (*3.4*)	24.0 (*1.3*)
Multipara	5	4
History of labor induction	0	0
History of labor augmentation	0	0
History of post-term delivery	0	0
Maternal history of post-term delivery		
None	4	4
Yes	0	0
Unknow	2	0
Paternal history of post-term delivery		
None	3	3
Yes	0	0
Unknow	3	1
Country of origin: Japan	6	4

**Table 3:** Outcome data collected following a pilot randomized controlled trial.

	Treatment Group (n = 6)	Usual Care Group (n = 4)
Labor induction (%)	0 (0)	0 (0)
Delivery week (*SD*)	39.8 (0.3)	40.1 (0.3)
Labor and delivery hours (*SD*)	6.0 (4.0)	3.3 (1.9)
Labor augmentation (%)	0 (0)	0 (0)
Delivery mode: spontaneous vaginal delivery (%)	6 (100)	4 (100)

## Data Availability

These data cannot be made publicly available since the participants did not provide their approval for the sharing of their data publicly. For researchers who meet the requirements for access to data, data are available from the corresponding author.

## References

[1] American College of Obstetricians and Gynecologists (2013). Definition of term pregnancy. Obstet. Gynecol..

[2] Swift E.M., Gunnarsdottir J., Zoega H., Bjarnadottir R.I., Steingrimsdottir T., Einarsdottir K. (2022). Trends in labor induction indications: A 20-year population-based study. Acta Obstet. Gynecol. Scand..

[3] Alfirevic Z., Kelly A.J., Dowswell T. (2009). Intravenous oxytocin alone for cervical ripening and induction of labour. Cochrane Database Syst. Rev..

[4] Suresh S.C., Kucirka L., Chau D.B., Hadley M., Sheffield J.S. (2021). Evidence-based protocol decreases time to vaginal delivery in elective inductions. Am. J. Obstet. Gynecol. MFM.

[5] Conell-Price J., Evans J.B., Hong D., Shafer S., Flood P. (2008). The development and validation of a dynamic model to account for the progress of labor in the assessment of pain. Anesth. Analg..

[6] Schwarz C., Gross M.M., Heusser P., Berger B. (2016). Women’s perceptions of induction of labour outcomes: Results of an online-survey in Germany. Midwifery.

[7] Kozhimannil K.B., Johnson P.J., Attanasio L.B., Gjerdingen D.K., McGovern P.M. (2013). Use of nonmedical methods of labor induction and pain management among U.S. women. Birth.

[8] Burns E., Blamey C., Ersser S.J., Lloyd A.J., Barnetson L. (2000). The use of aromatherapy in intrapartum midwifery practice an observational study. Complement. Ther. Nurs. Midwifery.

[9] Münstedt K., Brenken A., Kalder M. (2009). Clinical indications and perceived effectiveness of complementary and alternative medicine in departments of obstetrics in Germany: A questionnaire study. Eur. J. Obstet. Gynecol. Reprod. Biol..

[10] Weston M., Grabowska C. (2013). Complementary therapy for induction of labour. Pract. Midwife.

[11] Evans M. (2009). Postdates pregnancy and complementary therapies. Complement. Ther. Clin. Pract..

[12] Kaviani M., Maghbool S., Azima S., Tabaei M.H. (2014). Comparison of the effect of aromatherapy with Jasminum officinale and Salvia officinale on pain severity and labor outcome in nulliparous women. Iran. J. Nurs. Midwifery Res..

[13] Musil A. (2013). Labor encouragement with essential oils. Midwifery Today Int. Midwife.

[14] Tadokoro Y., Takahata K., Shuo T., Shinohara K., Horiuchi S. (2023). Changes in salivary oxytocin level of term pregnant women after aromatherapy footbath for spontaneous labor onset: A non-randomized experimental study. Int. J. Environ. Res. Public Health.

[15] Herz R.S. (2009). Aromatherapy facts and fictions: A scientific analysis of olfactory effects on mood, physiology and behavior. Int. J. Neurosci..

[16] Tadokoro Y., Horiuchi S., Takahata K., Shuo T., Sawano E., Shinohara K. (2017). Changes in salivary oxytocin after inhalation of clary sage essential oil scent in term-pregnant women: A feasibility pilot study. BMC Res. Notes.

[17] Tarumi W., Shinohara K. (2020). The Effects of essential oil on salivary oxytocin concentration in postmenopausal women. J. Altern. Complement. Med..

[18] Zahra A., Leila M.S. (2013). Lavender aromatherapy massages in reducing labor pain and duration of labor: A randomized controlled trial. Afr. J. Pharm. Pharmacol..

[19] Vakilian K., Keramat A. (2013). The effect of breathing technique with and without aromatherapy on the length of active phase and second stage of labour. Nurs. Midwifery Stud..

[20] Mansour Lamadah S. (2016). The effect of aromatherapy massage using lavender oil on the level of pain and anxiety during labour among primigravida women. Am. J. Nurs. Sci..

[21] Prevost M., Zelkowitz P., Tulandi T., Hayton B., Feeley N., Carter C.S., Joseph L., Pournajafi-Nazarloo H., Ping E.Y., Abenhaim H. (2014). Oxytocin in pregnancy and the postpartum: Relations to labor and its management. Front. Public Health.

[22] Feldman R., Weller A., Zagoory-Sharon O., Levine A. (2007). Evidence for a neuroendocrinological foundation of human affiliation: Plasma oxytocin levels across pregnancy and the postpartum period predict mother-infant bonding. Psychol. Sci..

[23] Takahata K., Horiuchi S., Tadokoro Y., Sawano E., Shinohara K. (2019). Oxytocin levels in low-risk primiparas following breast stimulation for spontaneous onset of labor: A quasi-experimental study. BMC Pregnancy Childbirth.

[24] Bowen D.J., Kreuter M., Spring B., Cofta-Woerpel L., Linnan L., Weiner D., Bakken S., Kaplan C.P., Squiers L., Fabrizio C. (2009). How we design feasibility studies. Am. J. Prev. Med..

[25] Eldridge S.M., Chan C.L., Campbell M.J., Bond C.M., Hopewell S., Thabane L., Lancaster G.A., PAFS Consensus Group (2016). CONSORT 2010 statement: Extension to randomised pilot and feasibility trials. Pilot Feasibility Stud..

[26] Thabane L., Ma J., Chu R., Cheng J., Ismaila A., Rios L.P., Robson R., Thabane M., Giangregorio L., Goldsmith C.H. (2010). A tutorial on pilot studies: The what, why and how. BMC Med. Res. Methodol..

[27] Seixas M.B., Ghisi G.L.M., Oh P., Pereira D.S., Moreira A.P.B., Jansen A.K., Batalha A.P.D.B., Cândido G.N., Almeida J.A., Pereira D.A.G. (2022). Feasibility of remote delivering an exercise and lifestyle education program for individuals living with prediabetes and diabetes in Brazil. Int. J. Environ. Res. Public Health.

[28] Oosterbos C., Rummens S., Bogaerts K., Van Hoylandt A., Hoornaert S., Weyns F., Dubuisson A., Ceuppens J., Schuind S., Groen J.L. (2023). A randomized controlled trial comparing conservative versus surgical treatment in patients with foot drop due to peroneal nerve entrapment: Results of an internal feasibility pilot study. Pilot Feasibility Stud..

[29] Matziorinis A.M., Flo B.K., Skouras S., Dahle K., Henriksen A., Hausmann F., Sudmann T.T., Gold C., Koelsch S. (2023). A 12-month randomised pilot trial of the Alzheimer’s and music therapy study: A feasibility assessment of music therapy and physical activity in patients with mild-to-moderate Alzheimer’s disease. Pilot Feasibility Stud..

[30] Hoefnagels J.W., Fischer K., Bos R.A.T., Driessens M.H.E., Meijer S.L.A., Schutgens R.E.G., Schrijvers L.H. (2020). A feasibility study on two tailored interventions to improve adherence in adults with haemophilia. Pilot Feasibility Stud..

[31] Andreassen M., Hemmingsson H., Boman I.L., Danielsson H., Jaarsma T. (2020). Feasibility of an intervention for patients with cognitive impairment using an interactive digital calendar with mobile phone reminders (RemindMe) to improve the performance of activities in everyday life. Int. J. Environ. Res. Public Health.

[32] Howell D.F., Malmgren Fänge A., Rogmark C., Ekvall Hansson E. (2023). Rehabilitation outcomes following hip fracture of home-based exercise interventions using a wearable device-A randomized controlled pilot and feasibility study. Int. J. Environ. Res. Public Health.

[33] Do C.K., Smith C.A., Dahlen H., Bisits A., Schmied V. (2011). Moxibustion for cephalic version: A feasibility randomised controlled trial. BMC Complement. Altern. Med..

[34] Lancaster G.A., Dodd S., Williamson P.R. (2024). Design and analysis of pilot studies: Recommendations for good practice. J. Eval. Clin. Pract..

[35] Posadzki P., Alotaibi A., Ernst E. (2012). Adverse effects of aromatherapy: A systematic review of case reports and case series. Int. J. Risk Saf. Med..

[36] Tisserand R., Young R. (2013). Essential Oil Safety: A Guide for Health Care Professionals. Churchill Livingstone.

[37] Leon A.C., Davis L.L., Kraemer H.C. (2011). The role and interpretation of pilot studies in clinical research. J. Psychiatr. Res..

[38] Orsmond G.I., Cohn E.S. (2015). The distinctive features of a feasibility study: Objectives and guiding questions. OTJR.

[39] O’Cathain A., Hoddinott P., Lewin S., Thomas K.J., Young B., Adamson J., Jansen Y.J., Mills N., Moore G., Donovan J.L. (2015). Maximising the impact of qualitative research in feasibility studies for randomised controlled trials: Guidance for researchers. Pilot Feasibility Stud..

[40] Choi P.T., Beattie W.S., Bryson G.L., Paul J.E., Yang H. (2009). Effects of neuraxial blockade may be difficult to study using large randomized controlled trials: The PeriOperative Epidural Trial (POET) Pilot Study. PLoS ONE.

[41] Kavanagh J., Kelly A.J., Thomas J. (2005). Breast stimulation for cervical ripening and induction of labour. Cochrane Database Syst. Rev..

[42] Smith C.A., Armour M., Dahlen H.G. (2017). Acupuncture or acupressure for induction of labour. Cochrane Database Syst. Rev..

[43] Sibbritt D.W., Catling C.J., Adams J., Shaw A.J., Homer C.S. (2014). The self-prescribed use of aromatherapy oils by pregnant women. Women Birth.

[44] Weijers M., Boumans N., van der Zwet J., Feron F., Bastiaenen C. (2023). A feasibility Randomised Controlled Trial as a first step towards evaluating the effectiveness of a digital health dashboard in preventive child health care: A mixed methods approach. Pilot Feasibility Stud..

[45] Cumming J., Clarke F.J., Holland M.J.G., Parry B.J., Quinton M.L., Cooley S.J. (2022). A feasibility study of the My Strengths Training for Life™ (MST4Life™) program for young people experiencing homelessness. Int. J. Environ. Res. Public Health.

[46] Pauley T., Percival R. (2014). Reducing post-dates induction numbers with post-dates complementary therapy clinics. Br. J. Midwifery.

[47] Oberg A.S., Frisell T., Svensson A.C., Iliadou A.N. (2013). Maternal and fetal genetic contributions to postterm birth: Familial clustering in a population-based sample of 475,429 Swedish births. Am. J. Epidemiol..

[48] Caughey A.B., Stotland N.E., Washington A.E., Escobar G.J. (2009). Who is at risk for prolonged and postterm pregnancy?. Am. J. Obstet. Gynecol..

[49] Mälstam E., Asaba E., Åkesson E., Guidetti S., Patomella A.H. (2023). The feasibility of make my day—A randomized controlled pilot trial of a stroke prevention program in primary healthcare. Int. J. Environ. Res. Public Health.

[50] Badaghi N., van Kruijsbergen M., Speckens A., Vilé J., Prins J., Kelders S., Kwakkenbos L. (2024). Group, blended and individual, unguided online delivery of mindfulness-based cognitive therapy for people with cancer: Feasibility uncontrolled trial. JMIR Form. Res..

